# Influence of Rectal Decompression on Abdominal Symptoms and Anorectal Physiology following Colonoscopy in Healthy Adults

**DOI:** 10.1155/2016/4101248

**Published:** 2016-08-29

**Authors:** Chih-Hsun Yi, Tso-Tsai Liu, Wei-Yi Lei, Jui-Sheng Hung, Chien-Lin Chen

**Affiliations:** Department of Medicine, Hualien Tzu Chi Hospital, Buddhist Tzu Chi Medical Foundation and Tzu Chi University, Hualien 97002, Taiwan

## Abstract

*Background*. Postcolonoscopy abdominal discomfort and bloating are common. The aim of this study was to evaluate whether rectal decompression improved distension-induced abdominal symptoms and influenced anorectal physiology.* Methods*. In 15 healthy subjects, rectal distension was achieved by direct air inflation into the rectum by colonoscopy. Placement of rectal and sham tube was then performed in each subject on a separate occasion. The anorectal parameters and distension-induced abdominal symptoms were recorded.* Results*. Anorectal parameters were similar between placements of rectal tube and sham tube except for greater rectal compliance with rectal tube than with sham tube (*P* < 0.05). Abdominal pain and bloating were significantly reduced by rectal tube and sham tube at 1 minute (both *P* < 0.05) and 3 minutes (both *P* < 0.05). After placement of rectal tube, abdominal pain at 3 minutes correlated positively with first sensation (*r* = 0.53, *P* = 0.04), and bloating at 3 minutes also correlated positively with urge sensation (*r* = 0.55, *P* = 0.03).* Conclusions*. Rectal decompression with either rectal or sham tube improved distension-induced abdominal symptoms. Our study indicates that the mechanisms that improved abdominal symptoms by rectal decompression might be mediated by a central pathway instead of a peripheral mechanism.

## 1. Introduction

Abdominal discomfort and bloating are frequently experienced by patients undergoing colonoscopy [1]. The most common symptom after colonoscopy is abdominal discomfort [2], which might cause an absence from work after colonoscopy [3]. There are some factors that have been thought to cause abdominal symptoms after colonoscopy, including the duration of the procedure, the degree of technical difficulty, distension due to air insufflations, and the expertise of the endoscopist. Excessive insufflation of air during the colonoscopy is the most important factor that causes abdominal discomfort due to the fact that many patients have difficulty expelling air after the procedure [4].

It has been demonstrated that anorectal sensory function is regulated by brain-gut interactions with a link to sensorimotor response of rectoanus during rectal distension [5]. It is yet unclear whether feeling of abdominal discomfort during rectal distension is physiologically influenced by direct rectal decompression, although we had shown that direct rectal suction helps relieve abdominal symptoms immediately after colonoscopy [6]. This study was designed to evaluate the effect of rectal decompression on abdominal symptoms following rectal air distension. The effect of rectal decompression on anorectal manometry and its relationships to abdominal symptoms were also determined.

## 2. Materials and Methods

### 2.1. Subjects

All subjects provided written informed consent and were interviewed about gastrointestinal symptoms prior to the studies. All participants did not have any history of underlying medical condition, previous gastrointestinal surgery, gastrointestinal symptoms, or clinical conditions affecting visceral perception. Subjects with poor communication or impaired hearing were excluded. We enrolled those subjects from a community and/or university population by public advertisement. The study protocol was reviewed and approved by the institutional review board of Hualien Tzu Chi Hospital, Buddhist Tzu Chi Medical Foundation. The study recruitment was between July 31, 2013, and May 21, 2014.

### 2.2. Anorectal Manometry

Subjects were instructed to evacuate the rectum and received Fleet's enema before the test. The probe was a 4.5 mm diameter, solid state catheter with multiple pressure transducers (Sandhill Scientific, Inc., Highlands Ranch, CO) and a lumen for balloon inflation. A 5 cm balloon was tied to the distal end of the catheter. The lubricated catheter was introduced into the rectum as patients lay in the left lateral position with their hips and knees flexed to 90°. Average resting and squeeze pressures (maximum and sustained) were recorded by the station pull-through technique. The threshold volume for rectoanal inhibitory reflexes (RAIR) was assessed by distending the rectal balloon in progressive 10 mL decrements, starting at 10 mL, until anal sphincter relaxation was observed at lower volume of distension. Rectal sensation was evaluated with rectal balloon inflated at an interval of 10 mL until the subject reported first sensation. The balloon volume was then increased by steps of 30 mL so that subjects experienced the sensations of urge to defecate as well as maximum distension. The threshold volumes for inducing these sensations were recorded. Rectal compliance for each balloon distention was derived from the slope of the volume-pressure curve.

### 2.3. Study Protocol and Design

All participants had bowel cleaning with ingestion of 90 mL of sodium phosphate followed by glycerin enema before the colonoscopy examination. Rectal distension was performed by air insufflations using a standard colonoscope (CF-240I, Olympus Optical Co. Ltd., Tokyo, Japan) with its tip placed near the rectosigmoid portion. Rectal distension was performed with progressive air insufflation until the subject felt maximal discomfort or unwillingness to commence further distension. Then, all participants underwent randomized placement with rectal tube and sham rectal tube (close with the tube ligated at the open end) with at least a week apart, which was placed into the rectum for 3 minutes (min) immediately after complete withdraw of the endoscope. The subjects had to complete anorectal manometry and abdominal symptoms immediately after withdraw of rectal tube or sham rectal tube.

### 2.4. Outcome Measurements

In all participants, abdominal pain and feeling of bloating were rated immediately after the procedure by using a Visual Analogue Scale (VAS, 0–100). A trained nurse evaluated the symptoms of the patient with VAS immediately after the procedure and 1 min and 3 min after withdraw of rectal tube or sham rectal tube. In addition, we measured the parameters of anorectal manometry including resting and squeeze sphincter pressure, sensory thresholds in response to balloon distension, compliance of rectum, and rectoanal inhibitory reflex in response to balloon distension in each subject, which were compared between rectal and sham rectal tube.

### 2.5. Statistical Analysis

Data were expressed as mean ± SEM. Statistical comparisons were assessed using Student's *t*-test or nonparametric test as appropriate. Correlations were studied with the Pearson test. A* P* value of <0.05 was considered to represent statistical significance.

## 3. Results

A total of 15 healthy subjects (5 women; mean age 25 years (range: 21–29)) completed 2 separate sessions in this study. All subjects tolerated the studies without any adverse effect. Baseline demographic characteristics are summarized in Table 1.

### 3.1. Postprocedural Abdominal Symptoms

**Figure 1:**
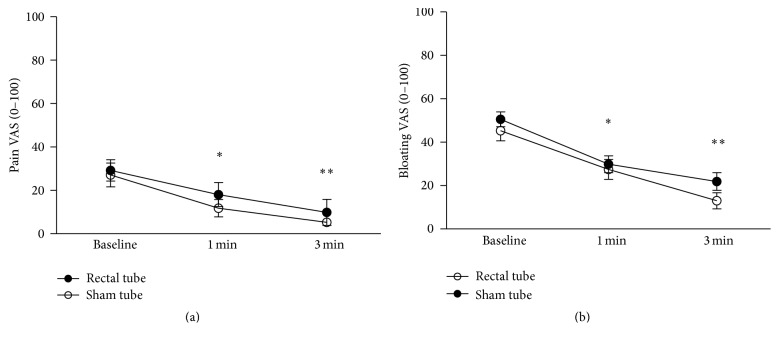
Abdominal symptoms after placement of rectal tube (a) and sham tube (b). After both treatments, abdominal pain and bloating significantly improved at 1 min and 3 min when compared with the baseline. ^*∗*^ *P* < 0.05, 1 min versus the baseline; ^*∗∗*^ *P* < 0.05, 3 min versus the baseline. Values are expressed as mean ± SEM.

When compared with symptoms at baseline, abdominal pain and bloating were significantly reduced by placement of rectal tube and sham tube at 1 min (both *P* < 0.05) and 3 min (both *P* < 0.05) (Figure 1). There was no difference for abdominal pain and bloating between the placements of rectal and sham tube throughout the examination (*P* = NS).

### 3.2. Anorectal Manometry Parameters

The rectal sensitivity for different levels of stimulation did not differ between placements of rectal tube and sham tube (*P* = NS) (Table 2). There was no group difference in the threshold volume for RAIR or anal sphincter length (*P* = NS) (Table 2). However, compliance was greater with rectal tube than with sham tube (*P* < 0.05) (Table 2). Anal sphincter pressure did not differ between placements of rectal tube and sham tube for resting, maximal, or sustained squeeze (*P* = NS) (Table 2).

### 3.3. Association between Abdominal Symptoms and Anorectal Manometry Parameters

**Figure 2:**
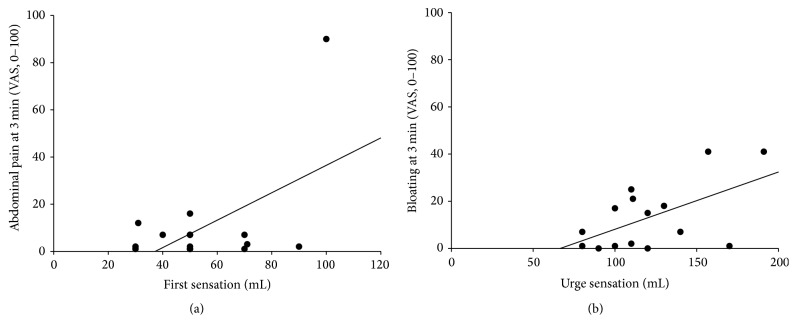
Association between abdominal symptoms and anorectal manometry after rectal tube treatment. Abdominal pain at 3 minutes correlates positively with first sensation (*r* = 0.53, *P* = 0.04) (a); bloating at 3 min correlates positively with urge sensation (*r* = 0.55, *P* = 0.03) (b). Values are expressed as mean ± SEM.

**Figure 3:**
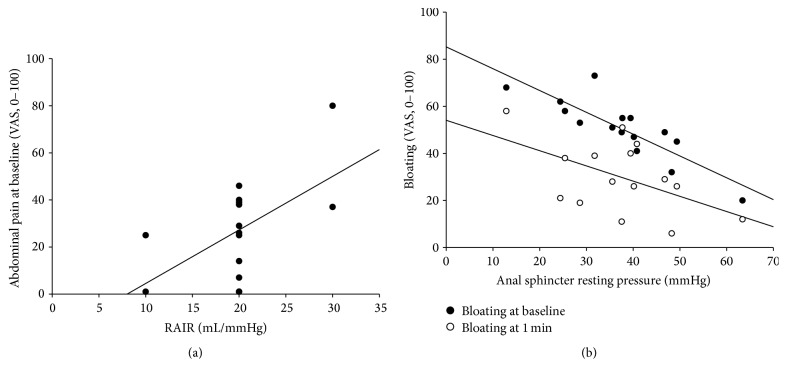
Association between abdominal symptoms and anorectal manometry after sham tube treatment. Baseline abdominal pain correlates positively with RAIR (*r* = 0.57, *P* = 0.03) (a). Anal resting pressure correlates negatively with baseline bloating (*r* = −0.85, *P* < 0.001) and bloating at 1 min (*r* = −0.52, *P* < 0.05) (b). Values are expressed as mean ± SEM. Line represents the mean value.

Abdominal pain at 3 min correlated positively with first sensation (*r* = 0.53, *P* = 0.04), whereas bloating at 3 min also correlated positively with urge sensation (*r* = 0.55, *P* = 0.03) after placement of rectal tube (Figure 2). Baseline abdominal pain correlated positively with RAIR (*r* = 0.57, *P* = 0.03) after placement of sham tube (Figure 3). Anal resting pressure correlated negatively with baseline bloating (*r* = −0.85, *P* < 0.001) and bloating at 1 min (*r* = −0.52, *P* < 0.05) after placement of sham tube (Figure 3).

## 4. Discussion

In this study, we investigated whether rectal decompression improved distension-induced abdominal symptoms. We have shown that abdominal symptoms were similarly improved with the placement of rectal tube and sham tube. Despite greater compliance with rectal tube placement, type of tube placement did not change the majority of anorectal function as measured by anorectal manometry. We observed some correlation between abdominal symptoms and anorectal sensation with the placement of rectal tube, while abdominal pain also correlated with RAIR with the placement of sham tube. In addition, we found a negative correlation between bloating and resting anal pressure with the placement of sham tube.

Previous studies have demonstrated that insertion of rectal tube at the conclusion of colonoscopy significantly improves abdominal symptoms and satisfaction with the procedure [7]. However, other studies did not observe beneficial effect in improving abdominal symptoms at the end of colonoscopy or 24 to 48 hours later [8]. The discrepancy in the findings among these studies can be explained by the fact that the rectal tube may decompress air in the rectum and sigmoid colon, which may not be adequate to reduce abdominal discomfort because most of the insufflated air may localize proximal to the sigmoid colon. Therefore, other studies with total decompression failed to find any improvement in abdominal symptoms after colonoscopy [9]. Other factors such as previous abdominal surgery with adhesion, technique difficulty, and high-pressure insufflations could not be eliminated simply with the decompression by the placement of rectal tube. In contrast to the assumption that insufflated air may present in the entire colon by the colonoscopy, the air we inflated is confined to rectum and sigmoid colon as we performed rectal distension only using a colonoscope with its tip placed near the rectosigmoid portion. Therefore, it is conceivable that we showed a significant improvement in abdominal pain and bloating with the placement of rectal tube.

In our study, abdominal symptoms were also improved by sham rectal tube. The actual explanation for such finding is unclear. However, it may be related to the phenomenon of “placebo effect” in which expectation of improvement can recruit common neurocircuitry along with brain processing [10]. Placebo reduction of affective responses to unpleasant stimuli is often underpinned by a decrease in central sensory processing [11]. The theoretical aspect of such explanation may be confirmed by functional neuroimaging studies which are beyond the scope of current work and hypothesis.

The mechanism through which rectal sensation, that is, first or urge sensation by anorectal manometry, links to abdominal symptoms of rectal distension in those subjects is yet unclear. It is well acknowledged that the rectum can perceive different types of stimuli via both thin myelinated A*δ* and unmyelinated C fibers in the rectal mucosa [12, 13]. In addition, rectal sensation can be activated, possibly evoked by stimulation of mechanoreceptors within the rectal wall and/or pelvic floor [14], which are stimulated by stretch-induced relaxation of the circular smooth muscle. Therefore, it has been indicated that a sensation of fullness, desire, or urgency to defecate can be consciously perceived depending on the degree of stretch relaxation [15]. Because feelings of abdominal symptoms during rectal distension as well as sensation for urge are related to conscious perception, it is probable that the afferent process for both stimuli is mediated by extrinsic rectal afferents (sympathetic and parasympathetic), conveying sensory information to higher brain center [16, 17]. The notion might also apply to the finding that abdominal pain correlates to RAIR, which is rectal distension threshold for the relaxation of the internal anal sphincter.

The reason why rectal compliance was greater with rectal tube than with sham tube is unclear. Decreased rectal compliance has been reported to be associated with rectal inflammation or increased rectal muscle tone and spasm [18]. Since rectal compliance is a measurement of “pressure-volume” relationship, it is likely that greater gas distension in the rectum with sham tube may impede expansion of rectal balloon for the measurement of rectal compliance.

In our study, we found a negative correlation between bloating and resting anal sphincter pressure. Resting pressures reflect the tonic activities of both the internal anal sphincter and the external anal sphincter, and approximately 75%–85% of the resting anal sphincter pressure is derived from the internal anal sphincter [19]. The actual explanation for the correlation between abdominal symptom and resting anal sphincter pressure is unclear. It is likely that lower resting anal sphincter pressure may allow easier passage of air through anal sphincter, by which bloating sensation is relieved quickly. However, further work needs to confirm this speculation.

In conclusion, we have shown that abdominal symptoms were significantly decreased by rectal decompression regardless of the type of rectal tube used (including sham tube). Despite the findings of the difference in rectal compliance and relationships among abdominal symptoms and anorectal parameters, most data of anorectal manometry were comparable between rectal and sham tubes. Our work indicates that the mechanisms by which abdominal symptoms get improved following rectal distension are more likely to be mediated by a central pathway rather than a peripheral mechanism. Therefore, a placebo effect in the modulation or attenuation of abdominal symptoms cannot be excluded. However, this notion needs to be confirmed by future studies with regard to the role of brain-gut interactions in the modulation of abdominal symptoms subsequent to rectal distension.

## Figures and Tables

**Table 1:** Demographic data in all subjects.

Variable	*n* = 15
Age (years)	25 (0.6)
Gender (female)	5 (33%)
Height (cm)	171 (1.8)
Weight (kg)	62.8 (2.6)
BMI (kg/m^2^)	21.3 (0.7)

Data are expressed as mean (SEM) or %.

**Table 2:** Anorectal function in all subjects after decompression.

Variable	Rectal tube	Sham tube
*Threshold volume (mL)*		
First sensation	55.5 (5.4)	52.3 (4.3)
Urge	120.6 (8.3)	114.7 (7.7)
Maximal	165.5 (9.9)	170.0 (10.0)
RAIR	20.7 (1.8)	20.0 (1.4)
*Anal sphincter pressure (mmHg)*		
Resting	42.0 (4.2)	37.4 (3.1)
Maximal	163.8 (28.1)	156.1 (25.0)
Sustained squeeze	204.7 (27.5)	192.5 (23.9)
Length of anal sphincter (mL)	1.9 (0.4)	2.0 (0.4)
Compliance (mL/mmHg)^*∗*^	11.2 (3.0)	4.0 (0.6)

Data are expressed as mean (SEM); ^*∗*^ *P* < 0.05.
